# Does the use of hernia mesh in surgical inguinal hernia repairs cause male infertility? A systematic review and descriptive analysis

**DOI:** 10.1186/s12978-018-0510-y

**Published:** 2018-04-23

**Authors:** Zhiyong Dong, Stacy Ann Kujawa, Cunchuan Wang, Hong Zhao

**Affiliations:** 1 Department of Surgery, the First Affiliated Hospital of Jinan University, No. 613. Huangpu Avenue West, Guangzhou, 510630 China; 2 Robert H. Lurie Comprehensive Cancer center, Division of Reproductive Science in Medicine, Feinberg School of Medicine, Northwestern University, 303 E. Superior Street, Suite 4-121, Chicago, IL 60611 USA

**Keywords:** Inguinal hernia repair, Mesh, Male infertility, Systematic review

## Abstract

**Objective:**

The aim of this study was to systematically review the available clinical trials examining male infertility after inguinal hernias were repaired using mesh procedures.

**Methods:**

The Cochrane Library, PubMed, Embase, Web of Science, and Chinese Biomedical Medicine Database were investigated. The Jada score was used to evaluate the quality of the studies, “Oxford Centre for Evidence-based Medicine-Levels of Evidence” was used to assess the level of the trials, and descriptive analysis was used to evaluate the studies.

**Results:**

Twenty nine related trials with a total of 36,552 patients were investigated, including seven randomized controlled trials (RCTs) with 616 patients and 10 clinical trials (1230 patients) with mesh or non-mesh repairs. The Jada score showed that there were six high quality RCTs and one low quality RCT. Levels of evidence determined from the Oxford Centre for Evidence-based Medicine further demonstrated that those six high quality RCTs also had high levels of evidence. It was found that serum testosterone, LH, and FSH levels declined in the laparoscopic group compared to the open group; however, the testicular volume only slightly increased without statistical significance. Testicular and sexual functions remained unchanged after both laparoscopic transabdominal preperitoneal hernia repair (TAPP) and totally extra-peritoneal repair (TEP). We also compared the different meshes used post-surgeries. VyproII/Timesh lightweight mesh had a diminished effect on sperm motility compared to Marlex heavyweight mesh after a one-year follow-up, but there was no effect after 3 years. Additionally, various open hernia repair procedures (Lichtenstein, mesh plug method, posterior pre-peritoneal mesh repair, and anterior tension-free repair) did not cause infertility.

**Conclusions:**

This systematic review suggests that hernia repair with mesh either in an open or a laparoscopic procedure has no significant effect on male fertility.

## Plain English summary

The incidence of inguinal hernia is steadily decreasing after the application of mesh and laparoscopic techniques; however the use of mesh causing infertility is becoming a growing concern. Whether there are any effects on male fertility after open/laparoscopic mesh inguinal hernia repair is still a controversial topic. Thus, the aim of this study was to systematically review the available clinical trials for male infertility after inguinal hernia repair with mesh. The Jada score and Oxford Centre for EBM Levels of Evidence were used to evaluate the quality or evidence level of the included studies. Finally, 29 related trials were investigated. The results indicated that polypropylene mesh inguinal repair did not change male infertility after open or laparoscopic mesh repair, TAPP versus TEP additional procedures of repair, or assorted mesh types. This study suggests that hernia repair with mesh either in an open or a laparoscopic procedure has no significant effect on male infertility according to current evidence. However, whether sperm should be stored and assessed for quality purposes prior to procedures for patients who have fertility issues, is worthy of further study.

## Background

Tension-free mesh hernia repair has become the standard procedure in inguinal hernia repair after the concept of tension-free hernia repair was proposed by Lichtenstein in 1989 [1]. Currently, the main operating procedures for inguinal hernia repair involve either open or laparoscopic hernia repair with mesh [2, 3]. The meshes used for these procedures are composed of biomaterial or biological material including polypropylene, Marlex, VyproII, TiMesh, and Prolene [4, 5]. The incidence of inguinal hernia has decreased after the application of mesh and laparoscopic techniques, but the use of mesh causing infertility is becoming a growing concern.

It has been reported that the complications of mesh hernia repair are infection, pain, adhesions, seroma, intestinal obstruction, and recurrence [6, 7]. Indicators for diagnosing male infertility usually include the testicular volume, testicular resistivity index, serum testosterone, serum gonadotrophins (FSH, follicle-stimulating and LH, luteinizing hormone), and semen quality (volume, concentration, motility, α-glucosidase, and morphology). Mesh inguinal hernia repair may cause infertility by influencing the spermatic duct structure in white male rats [8]. In men, 14 cases of azoospermia secondary to inguinal vasal obstruction were reported in relation to previous polypropylene mesh hernia repair [9]. A randomized controlled trial (RCT) with 59 male patients was used to evaluate male fertility between heavyweight meshes (Marlex) and lightweight meshes (Vypro II/TiMesh) at a one-year follow-up. Semen analysis showed that lightweight meshes for laparoscopic inguinal hernia repair negatively influenced sperm motility [10]. Contrarily, Tekatili et al. summarized 16 clinical studies and indicated that the lightweight mesh did not seem to have an impact on male fertility in inguinal hernias [11]. The only previous systemic review also supported that there is not an impact on male fertility after mesh hernia repairs [12]. Therefore, the role of mesh usage in male fertility in hernia repair patients remains unclear.

To circumvent the limitation of the previous review, we have included several additional RCTs and control trials on male infertility and hernia repair published from 2015 to 2017 [12–19], detailed sub-group analyses, and additional databases and clinical trials. In addition, the Jada score and levels of evidence from Oxford Centre for Evidence-based Medicine were used to assess the quality of included studies. Our most comprehensive systemic review analyzed the possible effect of mesh usage on male fertility in hernia repair, including different open and laparoscopic procedures and various types of surgical mesh. This study provides a robust evidence-based answer to support clinical decisions.

## Methods

### Search strategy

The related literature was searched on Feb 14th, 2018 from the following electronic databases: PubMed, Embase, CENTRAL (Cochrane Library), Web of Science, CBM (Chinese Biomedical Medicine Database), and other resources [WHOITRP (World Health Organization International Trials Registry Platform search portal, http://www.who.int/trialsearch/), ATCR (Australian New Zealand Clinical Trials Registry, http://www.anzctr.org.au/), ISRCTN (International Standard Randomized Controlled Trial Number Register, http://www.controlled-trials.com/), TC (Trials Central, www.trialscentral.org/), and CCTR (Chinese Clinical Trial Register, http://www.chictr.org.cn/)]. The following search strategy was used: (“polypropylene mesh” or “absorbable mesh” or “mesh” or “meshes”) and (“herniorrhaphy” or “hernioplasty” or “inguinal hernia repair” or “laparoscopic transabdominal preperitoneal hernia repair” or “totally extra-peritoneal repair”) and (“male infertility” or “fertility” or “azoospermia” or “sperm motility”). There were no language restrictions in this study.

### Inclusion and exclusion criteria

Clinical studies (RCTs, cohort studies, case controlled trials, case series, and case reports) were considered for this study. Review articles and letters to editors and unrelated papers were excluded. The study subjects were limited to men. The following outcomes were considered: testicular volume, testicular resistivity index, serum FSH, serum testosterone, serum LH, semen volume, α-glucosidase (mU), sperm morphology, sperm analysis (peak systolic velocity (PSV), end diastolic velocity (EDV), pulsatility index (PI), resistivity index (RI)), and sperm concentration.

### Data extraction and quality evaluation

All studies meeting the inclusion and exclusion criteria were retrieved by screening abstracts (DZ and WC). Two reviewers (DZ, WC) independently extracted the following terms by a self-made form generated from data included in each study: first author’s family name, publish year, country, type of surgery, study design, total number of patients, age, type of mesh, hernia side, outcomes, and the follow-up period. Any disagreements were resolved by joint discussion among reviewers, and the author was contacted if there was any missing data. The methodological quality of the included studies was assessed according to Jada scoring. The assess terms were: adequate sequence generation (0–2 points), allocation concealment (0–2 points), blinding (0–2 points), and follow-up/withdraw (0–2 points). For these assessments, 1 to 3 points were considered low quality and 4 to 7 points were deemed high quality. Methodological quality assessment was independently performed by two reviewers (DZ and WC) [20, 21]. The Oxford Centre for Evidence-based Medicine – Levels of Evidence (http://www.cebm.net/oxford-centre-evidence-based-medicine-levels-evidence-march-2009/) (Level I to Level V, level I was considered high level of evidence, level V was considered low level of evidence) was used to assess the level of the clinical trials.

### Statistical analysis

There was insufficient data included in the RCTs to perform the meta-analysis, so descriptive analysis was performed for these studies. The descriptive analysis was used if there was high clinical or statistical heterogeneity, and the subgroup analysis was used for high and low quality included studies or different interventions. The sensitivity analysis was performed when heterogeneity comes from the different methodological qualities of the included trials. Case control trials, cohort studies, retrospective, or case reports were also investigated by descriptive analysis. The egger’s test and Begg’s test were not used to explore the possibility of publication bias due to insufficient data included in the studies [22–26].

## Results

### Search strategy

**Fig. 1:**
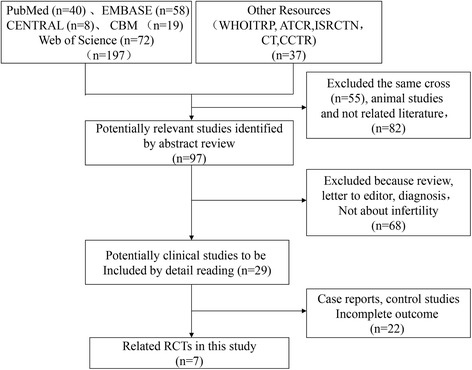
Flow diagram of the search process and study selection

A total of 234 studies were identified for screening via title and abstract according to our search strategy. Among them, 137 studies were excluded for the same cross-duplicated articles, animal studies, and unrelated literature. The remaining 97 potentially relevant studies were identified after screening by abstract, in which 68 studies were excluded because they were reviews, letters to the editor, diagnosis studies, and studies that did not focus on infertility. Consequently, the 29 clinical studies that met the inclusion criteria were included by full-text reading [27–48]. Among them, there were seven RCTs concerning mesh hernia repair and infertility [10, 13, 28, 31, 35, 44, 45]. Figure 1 displays the details of the search selection process.

### Study characteristics

This comprehensive systemic review focused on studies and reports published between 2003 and 2016 that investigated testicular function, semen, or male infertility after hernia repair, and it included 29 studies for a total of 36,916 participants. There were 15 studies conducted in Europe, 11 studies in Asia, and three in America. There were seven RCTs, eight case control studies, three cohort studies, three case series, four case reports, and three retrospective studies. The surgical operations included LAP (TAPP, TEP) and open (LHR). The main outcomes included: testicular volume, testicular resistivity index, serum FSH, serum testosterone, serum LH, semen volume, concentration, motility, α-glucosidase, morphology, peak systolic velocity, end diastolic velocity, pulsatility index, and obstructive azoospermia with a follow-up from six to 36 months. Table 1 demonstrates the characteristics of the included trials [9, 10, 13–19, 27–46].

**Table 1:** Baseline characteristics of the included studies in the systematic review

Study (Author/Year) (Evidence level)	Country	Surgery	Design	Patients (n)	Mean age (years)	Mesh materials	Hernia side	Outcome measure	Follow-up (months)
Bansal 2017 (I-1b) [13]	India	TAPP/TEP	RCT	TAPP 80	TAPP 40.9 ± 12.3	NS	Unilateral Hernia Bilateral hernia	Testicular functions Sexual functions	3
				VS TEP 80	TEP 40 ± 12.5				
Krnić 2016 (III-3b) [14]	Croatia	Open	Case-control	Non-complicated hernia 57 VS Incarcerated hernia 64	Group I 57 (40–81)	Bard® mesh	Right Hernia Left Hernia	Testicular blood flow	5
					Group II 64 (28–80)				
Lal 2016 (III-3b) [15]	India	TEP	Self Case-control	28	42.4 (18–72)	Bard 3DMax™ mesh	unilateral hernia (16 right sided and 8 left sided, 21 indirect and 3 direct hernias) and 4 to have bilateral hernia (2 direct and 2 indirect).	Resistive index	3
Gvenetadze 2016 (III-3b) [16]	Georgia	Open	Case control	Lichtenstein 66 Gvenetadze method 149	19–40	mesh	Bilateral	Oligospermia, reduction of the quantitative sperm	1,6
Shkvarkovskiy 2015 (III-3b) [17]	Russian	Open	Case control	New method 61	19–61	Polymeric mesh	Hernia	testicular arteries, testicular volume, sex hormones level.	NS
				Lichtenstein 63					
Yan 2015 (IV-4) [18]	China	Open	Retrospective study	142	24.0 ± 2.0	mesh	Unilateral	Infertility Sex function	3–36
Khodari 2015 (IV-4) [19]	France	Open	Retrospective study	69	NS	polypropylene mesh	Bilateral (history of hernia repair)	Risk of infertility	NS
Stula 2014 (II-2b) [27]	Croatia	TAPP/Open	Cohort study	TAPP 29	61(33–81)	Prolene mesh	Unilateral Bilateral	testicular blood flow (RI, PSV, EDV), ASA	5–6
				Open 53					
Peeters 2014 (I-1b) [28]	Belgium	TEP	RCT	Marlex ® 20	20–50	(Marlex ®) VyproII ® TiMesh ®	Unilateral 39 Bilateral 20	Semen analysis	36
				VyproII ® 20					
				TiMesh ® 19					
Schouten 2012 (protocol) [29]	Netherlands	TEP	Cohort study	21	18–60	Prolene	Bilateral inguinal hernias	testicular perfusion and volume, semen quantity and quality endocrinological status	6
Stula 2012 (II-2b) [30]	Croatia	TAPP/Open	Cohort	TAPP15 Open28	62(33–81)	Prolene mesh	Unilateral Bilateral	Testicular, capsular, intratesticular arterial flow dynamics	5
Singh 2012 (I-1b) [31]	India	TAPP, TEP/Open	RCT	LAP (TAPP, TEP)60 Open 57	LAP 45.7 ± 14.6	mesh	Unilateral Bilateral	Testicular functions (testicular volume, blood flow, hormones)	3
					Open 45.4 ± 17.8				
Hallén 2012 (IV-4) [32]	Sweden	Open	retrospective	Open 34,267	23–62	mesh	Unilateral Bilaterally	Risk for infertility	12
						No mesh			
Skawran 2011 (III-3b) [33]	Germany	TEP	Case control	Light mesh group 21	18–60	Bard TM soft mesh Bard TM flat mesh	Bilateral	Testicular volume and perfusion, serum levels of sexual hormones, ejaculate volume, and number of spermatic cells.	3
				Heavy group 38					
Hallén 2011 (III-3b) [34]	Sweden	NS	Case control	With mesh 232	18–55	mesh/ without mesh	Bilateral(344)	Risk for infertility	NS
				Without mesh 112					
				Control general 202					
Peeters 2010 (I-1b) [10]	Belgium	TEP	RCT	Marlex ® 20	20–50	(Marlex ®) VyproII ®	Unilateral 39 Bilateral 20	Semen analysis, scrotal ultrasonography	12
				VyproII ® 20		TiMesh ®			
				TiMesh ® 19					
Sucullu 2010 (I-1b) [35]	Turkey	open	RCT	Lichtenstein 32	LG 22 (20–28)	Polypropylene mesh	Unilateral	Testicular volume, resistive index Testicular function	3
				Mesh plug 32	MPG 23 (20–30)				
Kiladze 2009 (III-3b) [36]	Georgia	Open	Case control	Lichtenstein 56	44.8	mesh	Bilateral	Main sperm parameters	6
				Modified Lichtenstein 61					
Chu 2009 (IV-4) [37]	USA	Open	Case series	4	NS	mesh	NS	Testicular atrophy	6
Ramadan 2009 (III-3b) [38]	Turkey	Open	Case control	Indirect inguinal hernias 48	44.5 (30–73)	mesh	Unilateral direct inguinal hernia	testicular parenchyma, testicular arterial impedance, perfusion, venous flow	2
				Normal contralateral side 48					
Yamaguchi 2008 [39] (V-5)	Japan	Open	Case report	1	30	Polypropylene mesh	Bilateral herniorrhaphy	Hormonal testing Semen analyses Testicular size	15
Brisinda 2008 (IV-4) [40]	Italy	Open	Case series	26	NS	mesh	Hernia tension free repair	testicular perfusion	9
Dohle 2006 (V-5) [41]	Netherlands	open	Case report	2	35	Polypropylene mesh	Unilateral Bilateral	Semen analysis	3
Langenbach 2006 (I-1b) [42]	Germany	TAPP	RCT	Monofile, heavy-weight, rigid mesh 30 Smooth, heavy-weight variant of polypropylene 30 Polyglactin/polypropylene compound mesh 30	35–75	Rigid mesh Polypropylene Polyglactin/polypropylene	Unilateral	Testicular volume	3
Shin 2005 (IV-4) [9]	USA	LAP/Open	Case series	12 LAP 2 Open+ LAP	35.5 (28–42)	Polypropylene mesh	Unilateral Bilateral	Zaoospermia	6–36
Nagler 2005 (V-5) [43]	USA	Open	Case report	1	45	Polypropylene mesh	Right and left herniorrhaphy	Fructose-positive azoospermia	72
Akbulut 2003 (I-1b) [44]	Turkey	TEP / LHR	RCT	LHR 13 TEP 13	TEP 46.7 ± 1.7 LHR 54.2 ± 2.6	Polypropylene mesh	Unilateral Bilateral	Testicular function and volume	12
Aydede 2003 (I-1b) [45]	Turkey	Open	RCT	Posterior preperitoneal mesh repair 30 Anterior tension-free repair 30	22 > 60 38 < 60	mesh	Unilateral	Testicular flow spermatogenesis	2.5
Yang 1997 (V-5) [46]	China	Open (when child)	Case report	3	30/31/50	No mesh	Unilateral Bilateral	Obstructive azoospermia	30 year

*TAP* laparoscopic transabdominal preperitoneal hernia repair, *TEP* totally extra-peritoneal repair, *LHR* lichtenstein hernia repair, *LAP* laparoscopic hernia repair, *Group I* non-complicated hernia, *Goup II* incarcerated hernia. *NS* not state

### Quality assessment of the included studies

Table 2 displays the methodological quality of these studies according to Jada scores. Of all of the RCTs, seven studies reported adequate generation of the allocation sequence and one RCT provided unclear descriptions. Six trials reported allocation concealment [10, 13, 28, 35, 44, 45]. Blinding was not reported in any of the RCTs. Patients that were lost to follow-up or withdraw were reported in all studies. There were six studies that were considered high quality (5 points) and one RCT was low quality (3 points) according to Jada scores. The evidence of seven trials were level I_1b_, two were level II_2b_, and all of the RCTS were high level evidence.

**Table 2:** Quality assessment of RCTs studies (Jada Scores)

Study ID	Registration no.	Adequate sequence generation/scores(point)	Allocation concealment/scores(point)	Blinding/scores (point)	Follow-up/withdraw/scores(point)	Total Jada scores
Bansal 2017 [13]	CTRI009469	Yes(2)	Yes(2)	No(0)	Yes(1)	5
Peeters 2014 [28]	NCT00925067	Yes(2)	Yes(2)	No(0)	Yes(1)	5
Singh 2012 [29]	NS	Yes (2)	Yes(2)	No(0)	Yes(1)	5
Peeters 2010 [10]	NCT00925067	Yes (2)	Yes(2)	No(0)	Yes(1)	5
Sucullu 2010 [35]	NS	Yes(2)	Yes(2)	No(0)	Yes(1)	5
Akbulut 2003 [44]	NS	Yes (1)	Unclear(1)	No(0)	Yes(1)	3
Aydede 2003 [45]	NS	Yes (2)	Yes(2)	No(0)	Yes(1)	5

1–3 points considered as low quality; 4–7 points considered as high quality

### Descriptive analysis

There was high clinical heterogeneity among the included studies, so the meta-analysis was not used, and instead, the descriptive subgroup analysis was performed. The groups were divided into laparoscopic hernia repair groups and open hernia repair groups. Subgroups were divided into the following groups: mesh versus non-mesh, LAP versus Open, TAPP versus TEP, and Marlex mesh versus Vypro mesh. The detailed data from the outcomes of the seven RCTs is shown in Table 3.

**Table 3:** The detailed data from the outcomes of the seven RCTs

Study ID	Surgery	Total No. of patients		Outcome and data
		O	C	
Bansal 2017 [13]	TAPP VS TEP	80	80	Testicular volume: pre-operative, TAPP 13.1 ± 1.3, TEP 13.1 ± 1.2; 3 months, TAPP 13.1 ± 1.3, TEP 13.2 ± 1.1, 6 months TAPP 13.0 ± 1.3, TEP 13.2 ± 1.0 Testicular resistivity index: pre-operative, TAPP 0.64 ± 0.06, TEP 0.61 ± 0.07; 3 months, TAPP 0.634 ± 0.06, TEP 0.6 ± 0.07, 6 months TAPP 0.63 ± 0.06, TEP 0.6 ± 0.07 Serum FSH: pre-operative, TAPP 3.6 ± 0.8, TEP 3.4 ± 0.8; 3 months, TAPP 3.6 ± 1.0, TEP 3.4 ± 0.8, 6 months TAPP 3.6 ± 0.8, TEP 3.4 ± 0.9 Serum testosterone: pre-operative, TAPP 4.2 ± 1,TEP 4.0 ± 1.2; 3 months, TAPP 4.1 ± 0.9, TEP 4.0 ± 1.3, 6 months TAPP 4.1 ± 1.0, TEP 4.0 ± 1.2 Serum LH: TAPP 7.3 ± 1.1,TEP 7.3 ± 1.7; 3 months, TAPP 7.3 ± 1.0, TEP 7.3 ± 1.67, 6 months TAPP 7.4 ± 1.0, TEP 7.3 ± 1.6
Peeters 2014 [28]	Marlex® VS vyproII® Marlex® VS TiMesh®	12	15/10	3 year follow-up: Semen volume (ml): Marlex® -0.07 (− 1.1 to 0.6), vyproII® -0.1(− 1.5 to 0.2), TiMesh®-0.2 (− 0.9 to 1) Concentration (106 cells/ml): Marlex® -4.4 (− 16.1 to 0.5), vyproII® -5.5 (− 30.8 to 18.8), TiMesh®-1.65 (− 30.6 to 17.1) Motility (% progression): Marlex® -2.8 (− 18 to 4.3), vyproII® -8.5 (− 23 to 8.5), TiMesh®-8 (− 15 to − 4.5) a-glucosidase (mU): Marlex® 3.2 (− 15.5 to 6), vyproII® -5.5 (− 13.7 to 0.2), TiMesh® -1.4(− 8 to 1.75) morphology (% normal): Marlex® -2 (− 16 to 2), vyproII® -2.8 (− 9 to 0), TiMesh® -3 (− 8.5 to 4)
Singh 2012 [29]	Lap VS Open	60	60	Testicular volume: pre-operative, Lap, 9.8; Open 10.7; 3 month, Lap 9.3, Open 9.2 Resistitive index: pre-operative, Lap 0.64, Open 0.68; 3 month, Lap 0.58, Open 0.65 FSH: pre-operative, Lap 5,Open 5.1, 3 month, Lap 5.1, Open 6.1 LH: pre-operative, Lap 4.4,Open 4.5, 3 month, Lap 4.9, Open 5.4 Testosterone: pre-operative, Lap 5.7,Open 5.2, 3 month, Lap 5.5, Open 4.7
Peeters 2010 [10]	Marlex® VS vyproII® Marlex® VS TiMesh®	20	20/19	1 year follow-up: Semen volume (ml): Marlex® -0.05 (− 0.7 to 0.7), vyproII® -0.43 (− 1.3 to 0.3), TiMesh®0.2 (− 0.8 to 0.9) Concentration (106 cells/ml): Marlex® -9.6 (− 35.5 to 13), vyproII® -1.5 (− 21.5 to 10), TiMesh®2.1 (10.3 to 15.8) Motility (% progression): Marlex® -2.0 (− 2 to 10), vyproII® -9.5 (− 13.3 to − 1), TiMesh®-5.5 (− 17 to − 2) a-glucosidase (mU): Marlex® -3.6 (− 7.6 to 9.7), vyproII® -1 (− 3.7 to 3.7), TiMesh® 0(− 6.5 to 1.8) morphology (% normal): Marlex® 0 (− 4.3 to 5.8), vyproII® -1.8 (0 to − 5), TiMesh® -1.8 (− 6.8 to 5)
Sucullu 2010 [35]	LG VS MPG	32	32	Testicular volume: pre-operative, LG, 18.92 ± 1.05; MPG, 19.37 ± 1.06 3 months, LG 18.75 ± 1.26, MPG 18.21 ± 1.26 Resistive index: pre-operative,LG,0.64 ± 0.06, MPG 0.60 ± 0.04; 3 months, LG 0.80 ± 0.06, MPG 0.75 ± 0.08 Sperm concentration: pre-operative, LG,88.65 ± 10.30, MPG 75.27 ± 7.03; 3 months, LG 65.48 ± 8.22 MPG 58.87 ± 7.73 Rate of progressive motility: pre-operative, LG 52.79 ± 2.35, MPG 51.64 ± 2.60 3 months, LG 55.54 ± 2.26, MPG 48.53 ± 2.96
Akbulut 2003 [44]	TEP VS LHR	13	13	3-month. Testicular volume: pre-operative, TEP, 16.33 ± 0.71; LHR 15.44 ± 0.87; 3 month, TEP 16.70 ± 0.88, LHR 14.15 ± 0.96 FSH: pre-operative, TEP 6.47 ± 0.63, LHR 8.47 ± 1.11, 3 month, TEP 6.99 ± 0.86, LHR 9.12 ± 1.57 LH: pre-operative, TEP 4.06 ± 0.40, LHR 5.35 ± 0.57, 3 month, TEP 4.72 ± 0.70, LHR 5.64 ± 0.72 Testosterone: pre-operative, TEP 631.75 ± 60.52, LHR 544.48 ± 36.26, 3 month, TEP 672.00 ± 62.99, LHR 510.64 ± 39.71
Aydede 2003 [45]	TFR VS PPMR	30	30	peak systolic velocity (PSV):pre-operative TFR 11.1303 ± 0.6952, PPMR 10.25.20 ± 0.5033; 2.5 months, TFR 10.8400 ± 0.7084 PPMR 10.4890 ± 0.5194 end diastolic velocity (EDV): pre-operative TFR 3.1257 ± 0.1995, PPMR 3.0287 ± 0.5648; 2.5 months, TFR 1.4267 ± 6.544 PPMR 1.2957 ± 8.842 pulsatility index (PI): pre-operative TFR 1.3753 ± 9.177,PPMR 1.3460 ± 0.1082; 2.5 months,TFR 0.7193 ± 1.294 PPMR 0.6930 ± 1.887 resistivity index (RI): pre-operative TFR 0.6960 ± 2.192, PPMR 0.6867 ± 2.267; 2.5 months, TFR 2.8400 ± 0.1973 PPMR 3.0163 ± 0.1880

*O* observation group, *C* control group, *LG* Lichtenstein group, *MPG* Mesh plug group, *TFR* Anterior tension-free repair, *PPMR* Posterior preperitoneal mesh repair

### Laparoscopic mesh hernia repair group

There were 12 studies for a total of 1230 patients included in this group. The baseline characteristics are shown in Table 1.

### Sub-analysis

#### LAP (TAPP/TEP) versus open group

In the Singh 2012 study (Level I_1b_), there were a total of 117 patients with a mean age of 45.6 ± 16.2 years (range 17–79). In Group I, 32 patients underwent TEP and 28 underwent TAPP. Group II had 57 patients that underwent open mesh repair. The follow-up time was preoperatively and postoperatively set at 3 months. There was no significant difference between those two groups in testicular functions, preoperatively. There were statistically significant decreases in the testicular volume, preoperatively and postoperatively in the open group (*P* = 0.01), but there was no significant difference, preoperatively and postoperatively in the LAP group (*P* = 0.3). There was also statistical significance in the resistive index, preoperatively and postoperatively in the open group (*P* = 0.07) and the LAP group (*P* = 0.04). In the LAP group, there was no significant difference in FSH levels (*P* = 0.4) and testosterone (*P* = 0.3) between preoperatively and postoperatively; however the decrease was significant in LH levels (*P* = 0.01) after operation. In the open group, there was statistical significance in FSH (*P* = 0.01), LH (*P* = 0.001), and testosterone (*P* = 0.02) between preoperatively and postoperatively. This trial suggested that laparoscopic repair may be more suitable for preserving testicular functions [31]. In the Akbulut 2003 study (level I_1b_); there were a total of 60 patients with the age of 50.5 ± 4.4 (range 24–71). The follow-up time was 3 months. 26 patients were randomized and divided into the TEP group (13 patients) and Lichtenstein hernia repair (LHR) group (13 patients). There were no significant differences between preoperative and postoperative in both groups in regards to LH (*P* > 0.05) and FSH levels (*P* > 0.05). However, the decrease was significantly different in the testicular volume and testosterone levels in the TEP group (*P* < 0.05) compared to the LHR group (*P* > 0.05). It was indicated that the procedures would not alter LH, FSH, or testosterone values, but TEP could lead to a reduction in testicular volume within the normal limits [46]. Schouten 2012 designed a protocol for cohort studies in order to evaluate fertility after endoscopic TEP hernia repair, but no data has been published [29]. In Stula 2014 (II_2b_), there were a total of 543 patients with a mean age of 61 years (ranging 33–81). The follow-up time ranged from five to 6 months. There were 29 patients who underwent TAPP and 53 patients under open tension-free hernia repair. There was no significant difference between the two groups in baseline. The anti-sperm antibodies (ASA) value significantly increased in the open group after operation (*P* < 0.001), but there was no significant difference in the TAPP group (*P* = 0.133). There was significant change in the resistive index (*P* < 0.001) and capsular artery level (*P* = 0.02) of the resistive index (RI), in patients who underwent TAPP. End-diastolic velocity (EDV) showed significant differences on the testicular artery level (*P* = 0.032) in patients in the open group. This study showed that mesh hernia repairs, open or laparoscopically, caused only a transitory change in testicular blood flow, but there was no clinical significant difference [27]. In the Stula 2012 study (level II_2b_), there were a total of 43 patients with 62 years (range 33-81 years). The follow-up time was 5 months. There were 15 who underwent the TAPP procedure and 28 in the open (open tension-free hernia repair). This trial indicated that inguinal hernia mesh repairs do not have a clinical significant influence on testicular flow and sperm autoimmunity [30].

#### TAPP versus TEP group

In Bansal 2017 (level I_1b_), the RCT was divided into the TAPP group with 80 patients and TEP group with 80 patients. The mean age was 40.5 ± 12.4 (rang 18–60). The follow-up time was 3 months and 6 months. There was no significant difference in testicular volume (*P* > 0.05), testicular resistivity index (*P* > 0.05), FSH (*P* > 0.05), testosterone level (*P* > 0.05), and LH (*P* > 0.05) between the two groups at the 3 month or 6 month follow-up [13].

#### Different meshes comparable groups

In Peters’ 2010 study (level I_1b_), there were a total of 59 patients with an age range of 20–50 years. The patients were randomized into three groups: heavyweight polypropylene (Marlex®) with 20 patients, lightweight mesh (VyproII®) with 20 patients, and lightweight mesh (TiMesh®) with 19 patients, and all of the patients underwent TEP. The follow-up was at 1 year. This study suggests that the use of lightweight meshes for male patients with TEP could influence sperm motility (*P* = 0.013) at the 1 year follow-up [35]. In Peeters’ 2014 study (level I_1b_), he utilized the same patients as Peters’ 2010, but the follow-up time was increased to 3 years. There was decreased sperm motility after 1 year, but there was no significant difference among the three groups in semen volume (*P* > 0.05), concentration (*P* > 0.05), motility (*P* > 0.05), a-glucosidase (*P* > 0.05) and morphology (*P* > 0.05) after 3 years. In Langenbach’s 2006 study (evidence, level V), he mentioned a change in testicular volume, but there were no detailed data supporting the observation [28].

#### LAP group without controls

In this group, there were two studies: Lal 2016 (level III_3b_) and Skawran 2011 (level III_3b_). In the Lal 2016 study, there were a total of 28 patients: 24 with unilateral hernia, 4 with bilateral hernia who underwent TEP. The mean age was 42.4 years (range 18–72). The resistive index was followed-up at 24 h, 1 week, and 3 months and compared preoperatively against postoperatively. There was no significant difference in resistive indexes of testicular, capsular, and intratesticular arteries during any time postoperatively [15]. In the Skawran 2011 study, there were a total of 59 patients with an age range of 18–60 years who underwent a bilateral TEP repairs. In the prospectively (light mesh) group, there were 21 patients, the preoperative values were compared with postoperative values, and the follow-up time was 3 months. It showed that there were no statistical differences between preoperative and postoperative in testicular volume, testicular perfusion, FSH, LH, testosterone, and testicular function (ejaculate volume) (*P* > 0.05). There were 38 patients in the retrospective (heavy mesh) group where the follow-up was determined at ≥3 months. Again, there was no significant difference between the prospective group and retrospective group in testicular volume, testicular perfusion, FSH, LH, and ejaculate volume (*P* > 0.05) [33].

### Open mesh hernia repair group

#### Subgroup analysis

##### Compare with different hernia repair methods

In the Gvenetadze 2016 study (level III_3b_), there were a total of 215 patients with an age range from 19 to 40 years. 66 underwent bilateral Lichtenstein hernia repair and 149 underwent the bilateral Gvenetadze method (a modified Lichtenstein with spermatic cord isolation from a mesh by Gvenetadze. The follow-up times were set at 2 days prior to the operation, 30 days, and 6 months post operation. They found oligospermia and a 30–35% reduction of the quantitative sperm in the Lichtenstein group (*P* < 0.01). However, there was no significant difference in the Gvenetadze group [16]. In Shkvarkovskiy 2015 study (level III_3b_), there were a total of 124 patients with an age range from 19 to 61. 61 had their procedure with the new method (patent of Ukraine for useful model № 81,728) and 63 underwent the Lichtenstein hernia repair. The outcomes were testicular arteries, testicular volume, and the level of sex hormones. This study was published in Russian and supplied no detailed information [17]. We emailed the author but there was no response. In Sucullu’s 2010 study (level I_1b_), there were a total of 64 unilateral patients with an age range from 20 to 30 years. There were 32 patients in the Lichtenstein group and 32 underwent the mesh plug surgery. The follow-up time was 3 months. There was a significant increase in the RI in both the Lichtenstein group (*P* = 0.027) and the mesh plug group (*P* = 0.012), when comparing the preoperative with the postoperative values [35]. In Kiladze’s 2009 study (level III_3b_), there were a total of 117 bilateral patients with an average age of 44.8 years. The follow-up time was 6 months. 56 patients were in the Lichtenstein group and 117 were with the Gvenetadze group. Comparing the morphological parameters of sperm between the pre- and postoperative mesh hernia repair in these two groups, the results showed that complete isolation of the spermatic cord from the mesh prevents male infertility after a modified Lichtenstein hernioplast [36]. In Aydede’s 2003 study (level I_1b_), there were a total of 60 patients with 20 patients > 60 years old and 38 patients < 60 years old. 30 patients with posterior preperitoneal mesh repair (group I) with 30 patients were compared against the anterior tension-free repair (group II) with 30 patients. The follow-up time was pre-operative, early postoperative (the third day), and late postoperative (6 months). The results showed that there were significant differences between preoperative and early postoperative in Doppler flow parameters (spermatic cord and peak systolic velocity(PSV), end diastolic velocity(EDV), and resistivity index (RI)) (all *P* < 0.05). There was no significant difference between preoperative and late postoperative values in Doppler flow parameters [45].

##### Compare with different hernias

In Krnic’s 2016 study (level III_3b_), there were a total of 121 patients with an age range of 28–81 years. Group I had 57 patients with non-complicated hernia, and Group II had 64 patients with incarcerated hernia. Bard Mesh was used, and the follow-up time was 5 months. Resistive index, pulsative index, and antisperm temporarily fluctuated after the operation, but they returned to or were within normal values during the late postoperative phase in both groups. This study suggested that polypropylene mesh did not lead to any clinically significant complications on testicular flow in patients under open hernia repair with either non-complicated or incarcerated hernia [14]. In Ramadan’s 2009 study (level III_3b_), there were a total of 48 patients with indirect inguinal hernia, and the mean age was 44.5 years (range, 30–73 years). The contralateral non-hernia side was set as the control group. Testicular arterial impedance, venous plexus flow, and testicular perfusion were assessed pre-and postoperatively on both sides, and the follow-up time was 2 months. The results showed that there were no significant changes regarding testicular flow (*P* > 0.05) [38].

##### Compare with different meshes or no mesh

In Hallen’s 2012 study (level IV_4_), from 1992 to 2007, 34,267 men with an age range of 28 to 50 years, underwent an inguinal hernia repair involving at least one side. It was found that 57 of the 6281 men who underwent the unilaterally without mesh procedure were diagnosed with infertility. The observed cumulative incidence was 95% CI 0.91 (0.49–0.69) whereas the expected cumulative incidence was 1.03. There were 133 out of 22,420 men who underwent the unilaterally with mesh procedure that were diagnosed with infertility. The 95% CI of observed cumulative incidence was 0.59 (0.49 to 0.69), and the expected cumulative incidence was 0.67. In the operated bilaterally without mesh group, the infertility incidence was 0/226 where the expected cumulative incidence was 1.01. In operated bilaterally with mesh unilaterally group, the infertility incidence was 3/346, 95% CI of observed cumulative incidence was 0.87 (0 to 18.4), and the expected cumulative incidence was 1.05. In operated bilaterally with mesh on both sides, the infertility incidence was 19/2293, 95% CI 0.83 (0.46–1.20), and the expected cumulative incidence was 0.64, and in repeated repairs on any side, the values were 21/2701, 95% (0.45–1.11), and 0.68. The incidence of infertility had no significant change for either the mesh groups or the no-mesh groups. For most groups, the expected cumulative incidence was lower than the general population [32]. In Hallen’s 2011 study (level III_3b_), the study was based on data from the Swedish Hernia Register and questionnaire. There were a total of 525 participants analyzed. There were 232 in the bilateral mesh repair group with the mean age of 42.3 ± 8.8 years, 112 in the non-mesh group with 43.4 ± 8.8 years, and 181 in the normal population with 43.1 ± 8.1 years. There was no substantial effect in testicular status according to the questionnaire [34].

### Open hernia repair

In this group, these studies were either retrospective, case series, or case reports.

Yan et al. (level IV_4_) performed retrospective analysis for 142 young men under Lichtenstein, and the follow-up time was three to 36 months. There was no infertility found [18]. Khodari et al. (level IV_4_) reported that there were 69 azoospermia patients with a history of adult inguinal hernia repair surgery from 1990 to 2011, but there was no detailed report provided in the analysis [19]. Chu et al. examined four cases under the inguinal hernia mesh repair with the results showing that testicular ischemia of 2/4 patients was changed, caused by either the mesh loosening or being removed [37]. Yamaguchi et al. (level V_5_) reported that a 30 year old man had vas deferens obstruction after inguinal hernia repair with polypropylene mesh within several months [39]. Before azoospermia, men who underwent inguinal herniorrhaphy using polypropylene mesh needed to rapidly cryopreserve their sperm for future fertility; however Testicular / Epididymal Sperm Aspiration or Extraction (TESE-ICSI) was also a suitable treatment. Brisinda et al. (level IV_4_) prospectively analyzed 24 patients under open tension-free hernia repair with synthetic meshes in 2008 [40]. There were no statistically significant differences found in the testicular blood flow parameters and testicular volume comparing preoperative with postoperative. In fact, testicular flow improved in some cases. Dohle et al. (level V_5_) reported two cases of obstructive male infertility due to vassal obstruction after hernia repair with polypropylene mesh. It was believed that polypropylene mesh caused a dense fibroblastic reaction; thus affecting the vas deferens and spermatic cord [41]. Nagler et al. (level V_5_) reported a 45 year old man experienced obstructive azoospermia after polypropylene mesh repair and a left varicocelectomy. They thought that this issue was influenced by the mesh resulting in fibrosis of the vas deferens [43].

### Publication bias

Although there were seven RCTs in our study, there was no sufficient data included in the studies so the funnel plot, the egger’s test and Begg’s test were not explored.

## Discussion

### Laparoscopic mesh hernia repair group

#### LAP (included TAPP/TEP) versus open group

Singh et al. reported that there were significant decreases in testosterone, LH, and FSH with less growth in testicular volume under the laparoscopic group; however there was no significant difference in testicular atrophy in either the open repair with polypropylene mesh (heavyweight) or the laparoscopic inguinal hernia repair with polypropylene mesh groups [31]. Akbulut et al. reported that there was no significant difference in the TEP group or the Lichtenstein group in FSH, LH, testosterone, and testicular volume, but TEP may have decreased testicular volume post-operation to the normal limits with type I-b, II-a [44]. The diverse results with Singh et al. may have been caused by the small sample size, type of hernia, or possibly human error. For example, some TAPP procedures might have been mistakenly placed in the laparoscopic group. Stula et al. reported that mesh hernia repairs under open tension-free hernia repair or TAPP were only changed in the resistive index, end diastolic velocity, and peak systolic velocity in the early postoperative period but returned to a normal value, which they believe has no clinical significance. However, they did not compare the heavyweight against the light heavyweight; instead they only mentioned that the heavyweight mesh was used in the open hernia repair group. The age range included was from 17 to 81 years old, so the normal fluctuations might be related with age [27]. This result was similar to an earlier study published by Stula et al., in 2012 [30]. Overall, inguinal hernia mesh repair under open tension-free hernia repair or TAPP did not have clinical significance on testicular flow and immunological response. Thus, these results from the studies indicate that polypropylene mesh LAP inguinal repair did not alter male infertility during either procedure.

#### TAPP versus TEP group

Bansal et al. suggested that there was no change in testicular and sexual function after TAPP compared with TEP. According to the publication, changes in male infertility have no relation to the techniques used for TAPP or TEP; however they did not mention mesh in the procedures. Rather, the authors thought that handling the testicular vessels and cord structures during dissection may change the etiology of testicular dysfunction after open mesh repair [13].

#### Different meshes groups

Peeters et al. indicated that VyproII® or TiMesh® (lightweight mesh) decreased sperm motility when compared to Marlex® mesh (heavyweight) after a 1 year follow-up, but there was no significant difference after 3 years. In contrast, the lightweight mesh groups had a lower recurrence rate and no chronic pain, so lightweight mesh could be the superior choice [28, 35]. Junge et al. suggested that using modern low weight, large, porous, and elastic samples could have a benefit on the integrity of the vas deferens, when mesh is the required material to be used in younger patients undergoing open hernia repair [47].

#### LAP group, no control group

Lal et al. indicated that laparoscopic TEP operations do not alter testicular flow dynamics at 24 h, 1 week, or 3 months postoperative [15]. Skawarn et al. suggested that there was no evidence of impaired fertility after TEP operation with light or heavy mesh [33]. There was no case report found regarding infertility caused by the LAP procedure. The reason behind this is that the LAP procedure allows for less damage and stress to the spermatic cord.

### Open mesh hernia repair group

#### Compare with different hernia repair methods

Gvenetadze et al. indicated that the Gvenetadze method was better than Lichtenstein’s in preventing male infertility when undergoing open surgery [16]. Kiladze et al. suggested that the Gvenetadze method prevented male infertility though spermatic cord isolation from mesh in bilateral hernia procedures compared to the Lichtenstein hernia repair [37]. Contrarily, Sucullu et al. and Aydede et al. indicated that whether the Lichtenstein, mesh plug method, posterior pre-peritoneal mesh repair, or anterior tension-free repair were used, none of the procedures caused infertility. Thus, it seems that numerous hernia repair methods which are performed routinely in the clinic do not lead to infertility [35, 42, 45].

#### Compare different hernias

Krnic et al. reported that 57 patients had non-complicated hernia procedures whereas 64 patients with incarcerated hernia. Bard Mesh was used in both groups. This study suggested that polypropylene mesh did not lead to any clinically significant problems on testicular flow in patients undergoing open hernia repair with either non-complicated or incarcerated hernia [14]. In the Ramadan et al. study, it showed that there was no significant change to testicular flow in the hernia side vs. non-hernia side; thus, different types of hernias may not impact infertility under the open hernia repair with mesh [38].

#### Compare with meshes with no mesh

Hallen et al. performed an epidemiological survey in 2011, 232 in the bilateral mesh repair group and 112 in the non-mesh group were analyzed. There were no noteworthy effects in the testicular status of either group, according to the questionnaire. The authors believed that local legal circumstances and health care policies should be taken into consideration when doing sperm cryopreservation and the health care system should cover the cost of this fee if young men wish to have children later on in life [34]. The following year, Hallen et al. started a larger epidemiological survey where 0.9% (57/6281) of men were diagnosed with infertility after being operated on unilaterally without mesh compared to 0.59% (133/22420) of men with mesh. In the operated bilaterally, mesh on one side group, the infertility incidence was 0.87% (3/346). In operated bilaterally, mesh on both sides group, the incidence was 0.83% (19/2293) and in repeated repairs on any side, there was 0.77% (21/2701). The results showed that the incidence of infertility had no consequence in either the mesh groups with no-mesh groups, and mesh repair may continue to be used without major concern regarding the risk of male infertility [32].

#### Open hernia repair

In this group, these studies were retrospectives, case series, or case reports. Yan et al. and Brisinda et al. reported that there was no evidence that indicated that infertility was caused by the Lichtenstein or open tension-free hernia procedure [18, 40]. Khodari et al. mentioned that there were 69 azoospermia patients with a history of undergoing adult inguinal hernia repair surgery but did not describe the causes for the azoospermia [19]. Nagler et al. considered that a case with azoospermia was caused by mesh due to fibrosis of the vas deferens [43]. Yamaguchi et al. and Dohle et al. reported three cases with vas deferens obstruction after inguinal hernia repair with polypropylene mesh [39, 41]. Uzzo et al. reported in a 12 male beagle dog animal trial where half of the dogs were repaired using Marlex mesh and half had the classic Shouldice technique. There was a significant decrease in vasal luminal size with a marked soft tissue foreign body reaction identified in the Marlex mesh group [48].

Why could this change in procedure lead to infertility? There may be a relationship with mesh migration, surgeon skills, tightness of intraoperative suture or the surrounding tissue was not completely separated. The resulting postoperative bleeding, adhesions, and postoperative exercise frictions then trigger fibrosis which can lead to infertility. Chu et al. (level IV_4_) reported two cases of testicular ischemia that were altered under inguinal hernia mesh repair, caused by the mesh loosening or removal [37]. Although there are fewer reports on vas deferens blockage, we still should focus on the standardization for operative procedures in order to lessen and ultimately, eliminate postoperative complications from the treatment. Another reason behind the cause of these medical concerns could be due to the level of the surgeon’s skill. For example, if the blood vessels were damaged during the intraoperative operation, it would lead to vas deferens ischemia, which could cause infertility. Some cases that are diagnosed as infertility may be associated instead with the inguinal hernia. Singh et al. indicated that long term inguinal hernia patients might suffer from impairment in testicular blood flow, which could also lead to infertility [31]. Additional factors that could impact infertility could include the patient’s age, work status, psychological factors, and the environment. Previously, our animal studies suggested that the distribution of inguinal hernia may be related to estrogen levels, and these estrogen levels may be associated with infertility. Aydede et al. suggested that mesh repair is still a safe surgery in patients with no children or those who are currently undergoing infertility treatment [45]; but in our opinion, if a young man desires to have children in the future and is apprehensive about potential issues to his fertility due to the surgical procedure, he should have his semen examined and stored preoperatively to avoid future problems and medical disputes.

The major limitations of our study were the following: 1) there was high clinical heterogeneity between the included RCTs, 2) there were small samples for these included studies, and 3) the period of treatment for each study and mesh were different. For some of these studies, there was insufficient data provided for meta-analysis, and the evidence was weak, so funnel plot and meta-analysis were not performed. That will lead to some publication bias and less strong evidence. Larger samples, rigorous design, multi-center RCTs performed using diverse populations, and different mesh/intervention groups would be necessary to enhance this evidence and support a stronger conclusion regarding infertility in these procedures.

## Conclusion

The results of our review suggest that open or laparoscopic procedures with mesh hernia repair have no significant effects on male infertility according to the current RCTs and clinical trials (Evidence: level I). Overall, laparoscopic mesh repair might be more suitable to use for preserving testicular functions; however our main focus should be on standardizing operative procedures in order to lessen or eliminate postoperative complications.

## Abbreviations

ASA: Anti-sperm antibodies
ATCR: Australian New Zealand Clinical Trials Registry
CBM: Chinese biomedical medicine database
CCTR: Chinese clinical trial register
CENTRAL: Cochrane Library hosts the Central Register of Controlled Trials
EDV: End diastolic velocity
EDV: End-diastolic velocity
FSH: Follicle-stimulating hormone
ISRCTN: International Standard Randomized Controlled Trial Number Register
LH: Luteinizing hormone
LHR: Lichtenstein hernia repair
mU: α-glucosidase
PI: Pulsatility index
PSV: Peak systolic velocity
RCT: Randomized controlled trials
RI: Resistivity index
TAPP: laparoscopic transabdominal preperitoneal hernia repair
TC: Trials Central
TEP: Totally extra-peritoneal repair
TESE-ICSI: Testicular / Epididymal Sperm Aspiration or Extraction
WHOITRP: World Health Organization International Trials Registry Platform search portal
